# Determination of anodal tDCS intensity threshold for reversal of corticospinal excitability: an investigation for induction of counter-regulatory mechanisms

**DOI:** 10.1038/s41598-020-72909-4

**Published:** 2020-09-30

**Authors:** Maryam Hassanzahraee, Michael A. Nitsche, Maryam Zoghi, Shapour Jaberzadeh

**Affiliations:** 1 Non-Invasive Brain Stimulation and Neuroplasticity Laboratory, Department of Physiotherapy, School of Primary and Allied Health Care, Faculty of Medicine, Nursing and Health Science, Monash University, Melbourne, Australia; 2 Department of Neurology, University Medical Hospital Bergmannsheil, Bochum, Germany; 3 Department of Psychology and Neurosciences, Leibniz Research Centre for Working Environment and Human Factors, Dortmund, Germany; 4 Department of Rehabilitation, Nutrition and Sport, School of Allied Health, Discipline of Physiotherapy, La Trobe University, Melbourne, Australia

**Keywords:** Neuroscience, Synaptic plasticity

## Abstract

Transcranial direct current stimulation is applied to modulate activity, and excitability of the brain. Basically, LTP-like plasticity is induced when anodal tDCS (a-tDCS) is applied over the primary motor cortex. However, it has been shown that specific parameters of a-tDCS can induce a plasticity reversal. We aimed to systematically assess the intensity threshold for reversal of the direction of plasticity induced by a-tDCS, monitored by corticospinal excitability (CSE), and explored mechanisms regulating this reversal. Fifteen healthy participants received a-tDCS in pseudo-random order for 26 min with four intensities of 0.3, 0.7, 1, and 1.5 mA. To measure CSE changes, single-pulse TMS was applied over the left M1, and motor evoked potentials of a contralateral hand muscle were recorded prior to a-tDCS, immediately and 30-min post-intervention. Paired-pulse TMS was used to evaluate intracortical excitation and inhibition. CSE increased significantly following a-tDCS with an intensity of 0.7 mA; however, the expected effect decreased and even reversed at intensities of 1 and 1.5 mA. ICF was significantly increased while SICI and LICI decreased at 0.7 mA. On the other hand, a significant decrease of ICF, but SICI and LICI enhancement was observed at intensities of 1, and 1.5 mA. The present findings show an intensity threshold of ≥ 1 mA for 26 min a-tDCS to reverse LTP- into LTD-like plasticity. It is suggested that increasing stimulation intensity, with constant stimulation duration, activates counter-regulatory mechanisms to prevent excessive brain excitation. Therefore, stimulation intensity and plasticity induced by a-tDCS might non-linearly correlate in scenarios with prolonged stimulation duration.

## Introduction

Anodal transcranial direct current stimulation (a-tDCS) is a non-invasive brain stimulation tool which induces long-term potentiation (LTP)-like plasticity of the human brain via application of weak direct currents, it enhances corticospinal excitability (CSE)^1^. Within certain limits, the respective stimulation effect depends linearly on the intensity (up to 1 mA) and duration (up to 13 min) of a-tDCS, as shown by earlier studies^1–3^. This finding has been supported by a number of studies stating polarity-dependent excitatory effects of a-tDCS on CSE^4–7^, and more recent studies, which explored an extended range of stimulation intensity and duration^8,9^. TMS-EEG studies offer valuable adjunctive information, because they allow for a relatively specific measure of cortical excitability. Indeed, a couple of respective studies have shown excitability-enhancing effects of anodal tDCS, which is in agreement with relevant cortical effects of this intervention^10–13^.

The assumption of a generally linear association between stimulation intensity/duration and LTP-like plasticity was however challenged by other studies, which showed a reduction or even reversal of tDCS-induced excitability alterations with specific current intensities and/or stimulation durations^9,14–19^. Other a-tDCS studies have shown no significant CSE-difference following different stimulation intensities for stimulation durations of 10 min^20,21^, 15 min^8^, 20 min^9,15^, and 30 min^9^. These findings suggest a more complex interaction between stimulation parameters, and the induced plasticity, as already shown for cathodal tDCS^15,22^. The exact boundary conditions of respective effect reversals, and non-linearities, as well as mechanisms, have however not been explored in detail. These could be caused by counter-regulatory effects which might be driven by alterations of GABAergic, and glutamatergic feedback loops, and involve calcium-dependent mechanisms, including the activation of potassium channels induced by calcium overflow^14,23,24^. In accordance, reduction of calcium influx by pharmacological block of voltage-gated calcium channels abolished the conversion of LTP- to LTD-like plasticity induced by a 26 min/1 mA a-tDCS protocol^14^. Moreover, higher intensity, and longer duration of stimulation might enhance calcium influx in a larger number of neurons to a sufficient degree to induce plasticity, which could explain why beyond reversal of the after-effects of stimulation, with even longer, and/ or stronger stimulation the primary plasticity effect—in case of a-tDCS LTP-like plasticity—can re-emerge^9^.

In our foregoing study, we have systematically explored the critical stimulation duration required for reversal of the after-effects of a-tDCS, and kept current intensity constant^19^. The results revealed a duration threshold for reversal of the excitability-enhancing effect of a-tDCS with stimulation durations ≥ 26 min^19^. In the present study we were interested to explore if beyond duration also the intensity of stimulation is relevant for the reversal of a-tDCS after-effects. Based on the calcium-dependency hypothesis, we expected an intensity-dependency for reversal of tDCS after-effects, with only higher stimulation intensities (based on the results of previous studies equal or above 1 mA^14,20^) reversing LTP- into LTD-like plasticity. In addition, we aimed to investigate neurotransmitter-dependent mechanisms responsible for this reversal effect. We hypothesised that with higher stimulation intensities, which were expected to cause a reversal of CSE, intracortical facilitation driven by glutamatergic *N*-methyl-d-aspartate (NMDA) receptors would be reduced, while gamma-aminobutyric acid (GABA)-dependent cortical inhibition would be enhanced.

## Results

All participants completed all experimental sessions and tolerated all experimental conditions well. The Shapiro–Wilk test confirmed normality of all data sets. The respective one-way rmANOVAs revealed no significant difference between baseline values of MEP amplitudes (sp- and pp-MEPs) (Table 1), and baseline and post-intervention TMS stimulus intensity (SI_1mV_) (Table 2) between the respective sessions with different current intensities. There was also no significant difference between the baseline and post-intervention single pulse MEPs for paired-pulse protocols (P > 0.05).

**Table 1:** Baseline TMS measurements.

Baseline Measurements	Experimental conditions (a-tDCS intensities				df	F value	P value
	0.3 mA	0.7 mA)	1 mA	1.5 mA			
CSE (mV)	1.06 ± 0.05	1.09 ± 0.04	1.04 ± 0.02	1.08 ± 0.06	3	1.62	0.19
SICI (%)	33.59 ± 1.32	30.04 ± 1.54	35.60 ± 1.03	32.87 ± 2.24	3	0.51	0.67
ICF (%)	115.70 ± 3.21	119.10 ± 2.90	113.73 ± 3.05	117.83 ± 4.72	3	2.46	0.10
LIF (%)	119.92 ± 6.09	111.83 ± 4.12	110.62 ± 3.99	114.76 ± 5.90	3	0.31	0.76
LICI (%)	30.36 ± 3.56	30.29 ± 1.96	31.13 ± 1.69	28.39 ± 2.34	3	0.61	0.63

Mean ± Standard error of mean (SEM). A one-way ANOVA was calculated for inter-session differences of the average baseline CSE (1 mV), SICI, LICI, ICF, and LIF. There was not significant difference across experimental sessions for all baseline measurements. *CSE* corticospinal excitability, *SICI* short-latency intracortical inhibition, *ICF* Intracortical facilitation, *LIF* long interval facilitation, *LICI* long-latency intracortical inhibition.

**Table 2:** TMS Stimulus intensity (in percentage MSO) of baseline and post-intervention (mean ± SEM).

Experimental conditions	TMS stimulus intensity (%)					
	RMT		P value	SI_1mV_		P value
	Baseline	Post-intervention		Baseline	Post-intervention	
**A-tDCS intensities**						
0.3 mA	37.46 ± 1.17	37.13 ± 1.32	0.49	46.13 ± 2.88	45.33 ± 1.54	0.017*
0.7 mA	38.66 ± 1.37	36.53 ± 1.59	0.001*	47.21 ± 2.66	45.28 ± 1.38	0.004*
1 mA	37.60 ± 1.49	38.40 ± 1.35	0.25	46.87 ± 1.81	48.04 ± 1.72	0.008*
1.5 mA	38.13 ± 1.08	40.26 ± 1	0.001*	45.12 ± 2.98	47.85 ± 1.31	0.001*
P value	0.87	0.30		0.82	0.2	

There was not significant difference across experimental sessions for baseline measurements. *SI1mV* stimulus intensity (for an average motor evoked potential (MEP) of 1 mV), *RMT* resting motor threshold. (*) shows significant difference.

### The effects of a-tDCS intensity on CSE

**Figure 1:**
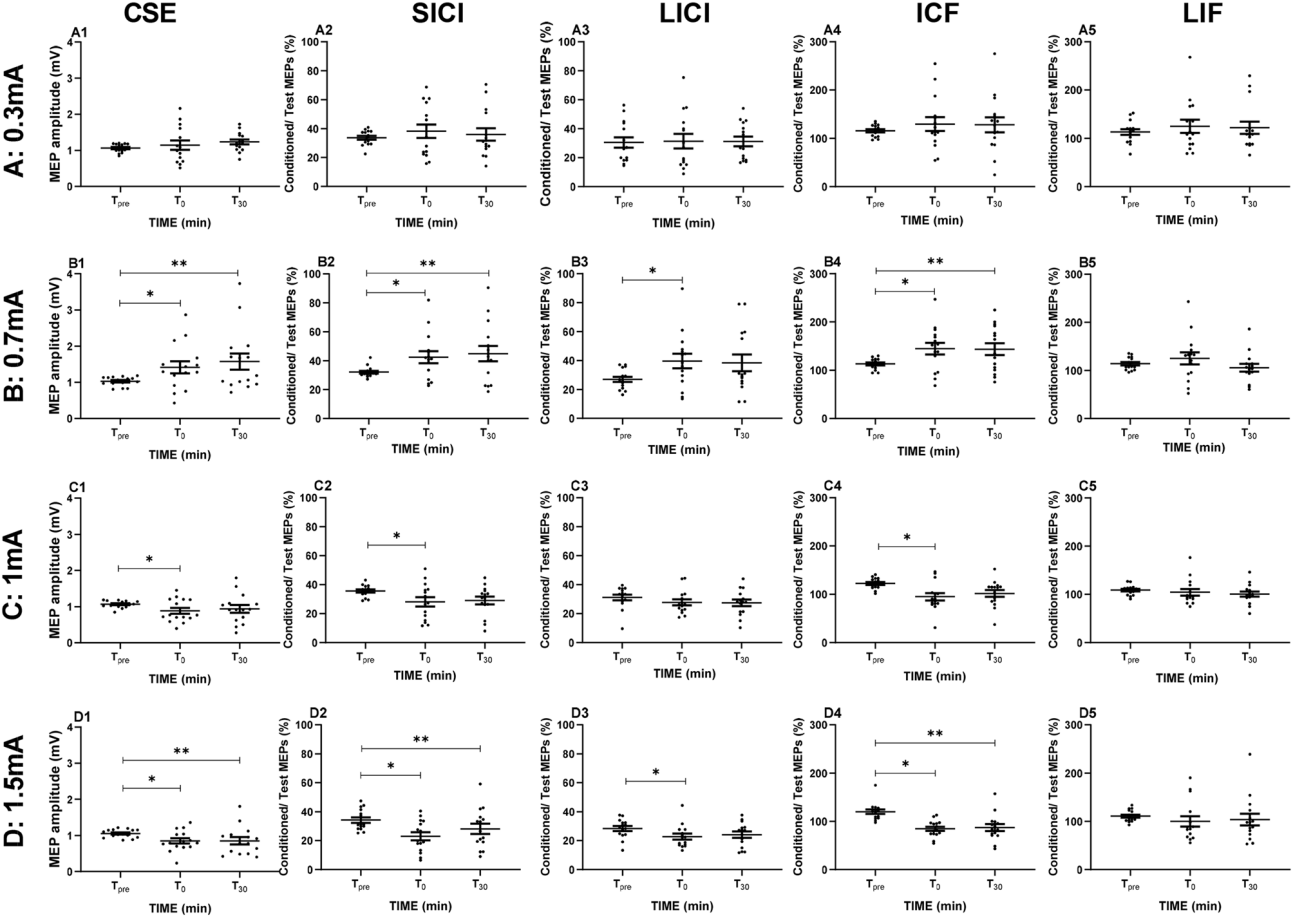
The effects of different intensities of a-tDCS on corticospinal excitability (CSE; **A1**–**D1**), short latency intracortical inhibition (SICI; **A2**–**D2**), long latency intracortical inhibition (LICI; **A3–D3**), intracortical facilitation (ICF; **A4–D4**), and long interval facilitation (LIF; **A5–D5**). **A1–A5**: 0.3 mA, **B1–A5**: 0.7 mA, **C1–C5**: 1 mA, **D1–D5**: 1.5 mA. (*) shows significant differences between baseline (T_pre_) and T_0_, T_30_ (P < 0.05). CSE and ICF were enhanced at a stimulation intensity of 0.7 mA, and decreased at 1 and 1.5 mA. In contrast, SICI and LICI decreased at 0.7 mA and increased at 1 and 1.5 mA. Each dot represents one participant. Lines show the means. Error bars represent standard error of the mean (SEM).

The two-way rmANOVA showed a significant main effect of ‘intensity’ (F_(3, 42)_ = 9.77, P > 0.01, η_p_^2^ = 0.48) and a significant ‘intensity × time’ interaction (F_(6, 84)_ = 4.69, P < 0.01, η_p_^2^ = 0.25). The Bonferroni-corrected post-hoc analysis revealed significant differences for tDCS intensities of 0.3 vs. 1.5 mA (P = 0.01), and 0.7 vs. 1.0 (P = 0.01), and vs. 1.5 mA (P = 0.007). The peak-to-peak MEP amplitudes significantly increased (T_0_, T_30_) following a-tDCS with 0.7 mA, as compared to baseline. On the other hand, MEP amplitudes showed a significant reduction following tDCS intensities of 1 (T_0_) and 1.5 mA (T_0_, T_30_), as compared to T_pre_. Figure 1A1–D1 shows CSE changes of all participants for the four a-tDCS intensities.

### The effects of a-tDCS intensity on SICI and LICI

The two-way rmANOVA revealed a significant main effect of ‘intensity’ (F_(3, 42)_ = 4.65, P < 0.01, η_p_^2^ = 0.28), and the ‘intensity × time’ interaction on SICI (F_(6, 84)_ = 4.27, P < 0.01, η_p_^2^ = 0.23). Pairwise comparisons revealed significant differences for tDCS intensities of 0.7 vs. 1.5 mA (P = 0.009). SICI decreased significantly following stimulation with 0.7 mA (T_0_, T_30_), while it increased significantly following stimulation intensities with 1.0 (T_0_), and 1.5 mA (T_0_, T_30_), as compared to baseline values (Fig. 1A2–D2). The rmANOVA conducted for LICI showed a significant main effect of ‘intensity’ (F_(3, 42)_ = 2.75, P < 0.05, η_p_^2^ = 0.23), and the ‘intensity × time’ interaction (F_(6, 84)_ = 2.30, P < 0.05, η_p_^2^ = 0.32). LICI decreased significantly following tDCS with an intensity of 0.7 mA (T_0_), while it increased significantly following tDCS intensity of 1.5 mA (T_0_), as compared to baseline measures (Fig. 1A3–D3).

### The effects of a-tDCS intensity on ICF and LIF

The rmANOVA conducted for ICF showed a significant main effect of ‘intensity’ (F_(3, 42)_ = 6.10, P < 0.01, η_p_^2^ = 0.32) and ‘intensity × time’ interaction (F_(6, 84)_ = 6.09, P < 0.001, η_p_^2^ = 0.30). Pairwise comparisons revealed significant differences for tDCS intensities of 0.7 vs. 1.0 mA (P = 0.01), and 0.7 vs. 1.5 mA (P = 0.001). ICF increased significantly after tDCS with 0.7 mA (T_0_, T_30_), but was significantly reduced after stimulation with intensities of 1.0 (T_0_), and 1.5 mA (T_0_, T_30_) (Fig. 1A4–D4). The rmANOVA conducted for LIF showed no significant main and interaction effects (P > 0.05; Fig. 1A5–D5).

### Safety and side effects of A-tDCS

No side and/ or adverse effects were reported after a-tDCS, except tingling and light itching under the electrodes during stimulation reported by some of the participants in all experimental conditions. No burning sensations, headaches, or pain were recorded during or after stimulation. Side effect means ± SEM are reported in Table 3, and did not differ significantly between sessions (P > 0.05).

**Table 3:** Side effects are based on ratings on a Numeric Analogue Scale (NAS).

Side effect	Anode (target electrode)				Cathode (return electrode)			
	0.3 mA	0.7 mA	1 mA	1.5 mA	0.3 mA	0.7 mA	1 mA	1.5 mA
**Tingling sensation**								
Beginning	2.7 ± 0.14	3.9 ± 0.23	4.3 ± 0.48	4.8 ± 0.24	2.3 ± 0.21	2.7 ± 0.09	2.1 ± 0.26	2.6 ± 0.11
Middle	2.4 ± 0.23	2.3 ± 0.14	2.7 ± 0.19	2.6 ± 0.22	0.8 ± 0.15	1.0 ± 0.10	1.0 ± 0.21	1.2 ± 0.17
End	1.7 ± 0.15	1.3 ± 0.21	1.8 ± 0.22	2.0 ± 0.19	0.5 ± 0.21	0.6 ± 0.12	0.4 ± 0.18	0.6 ± 0.2
**Itching sensation**								
Beginning	2.7 ± 0.27	2.6 ± 0.17	3.1 ± 0.28	3.7 ± 0.21	1.8 ± 0.13	1.9 ± 0.10	2.2 ± 0.16	2.7 ± 0.27
Middle	1.8 ± 0.13	1.5 ± 0.11	2.1 ± 0.28	2.2 ± 0.12	1.1 ± 0.18	1.4 ± 0.1	1.1 ± 0.25	1.5 ± 0.18
End	0.9 ± 0.11	1.2 ± 0. 12	1.0 ± 0.19	1.1 ± 0.12	0.9 ± 0.08	0.8 ± 0.10	0.6 ± 0.11	0.9 ± 0.09
**Burning sensation**								
Beginning	–	–	–	–	–	–	–	–
Middle	–	–	–	–	–	–	–	–
End	–	–	–	–	–	–	–	–
**Not tolerated**								
Beginning	–	–	–	–	–	–	–	–
Middle	–	–	–	–	–	–	–	–
End	–	–	–	–	–	–	–	–

0 is representing no sensation, and 10 as the worst sensation imaginable. The sensations were recorded during three phases of stimulation: Beginning (0 min to 1/3 of stimulation duration), Middle (1/2 to 2/3 of stimulation duration), End (last 6 min to end of stimulation). Sensations under both, target (anode) and return (cathode) electrodes were recorded. Scores are reported as mean ± SEM. (–) indicates that no sensations were reported.

The Chi-square test conducted to control for blinding showed no significant differences between conditions [χ^2^ (3, n = 15) = 5.37, P = 0.09]. This result demonstrates that participants were unable to distinguish between the experimental conditions in this study. The percentage of participants who replied ‘No’ was 89%. Therefore, the blinding procedure was successful in the present study.

## Discussion

The results of the current study suggest the existence of an intensity threshold for the reversal of the excitability-enhancing effects of a-tDCS applied for 26 min on CSE. This finding confirms that of our previous study on a stimulation duration threshold^19^ in which the excitability effects of 1 mA a-tDCS reversed at stimulation durations ≥ 26 min. Accordingly, unlike a-tDCS at lower intensities (< 1 mA), higher intensities (≥ 1 mA, 26 min) reversed the excitatory effects of a-tDCS on CSE. The results of the present study also confirm that the reversal of CSE at higher intensities (≥ 1 mA) is associated with specific alterations of intracortical physiology, including an increase of inhibitory and decrease of excitatory mechanisms. TDCS was well tolerated in all experimental sessions, and the blinding procedure was successful.

### The effects of A-tDCS intensity on CSE and Intracortical excitability

#### 26 min a-tDCS with intensities < 1 mA

We hypothesized that a-tDCS with intensities < 1 mA would increase CSE and assumed that this increase would be accompanied by reduction of SICI and/or LICI, an increase of ICF and/or LIF. Our findings partially support these hypotheses. Indeed, our results showed that CSE was significantly increased at a-tDCS of 0.7 mA in line with the results of previous studies using a-tDCS < 1 mA^1,8,20,21,38^. However, the missing increase of CSE following a-tDCS of 0.3 mA was in contrast with studies of Bastani and Jaberzadeh (0.3 mA)^38^, and Chew et al. (0.2 mA)^21^, probably related to smaller electrodes used in those studies (24 cm^2^ and 16 cm^2^) compared to the present study (35 cm^2^), which result in larger current densities. The findings of the present study show furthermore a reduction of SICI and LICI following 0.7 mA a-tDCS, confirming reduced inhibition associated with excitability-enhancing a-tDCS shown in some previous studies^20,34,39,40^. Furthermore, the results reveal an increase of ICF and LIF, in accordance with our hypothesis, and other studies^15,34^.

The findings of the paired-pulse protocols show thus an increase in intracortical facilitation, and decrease in inhibition in case of excitability-enhancing effects of tDCS. The increase in ICF supports the main involvement of calcium-dependent mechanisms, since ICF is mainly controlled by glutamatergic NMDA receptor activity, which has calcium channel properties^3,41–44^. The observed disinhibition shown by reduced SICI, and LICI suggests the presence of a gating effect by reduced GABA activity, which controls for these TMS parameters^3,34,40,45^.

#### 26 min a-tDCS with intensities ≥ 1 mA

We hypothesized that the facilitatory effect of a-tDCS on CSE would decrease and even reverse into LTD-like plasticity when stimulation intensities of ≥ 1 mA are used. Our results support this hypothesis, and are in line with previous studies showing non-linear effects of a-tDCS^8,14,17–19^. On the other hand, these findings are not in line with other studies showing a CSE increase following higher intensities of stimulation of 1.2, 1.5, and 2 mA probably due to shorter stimulation duration (10 min) compared to the current study^20,21,38^. We also hypothesized that specific intracortical excitability changes would accompany the CSE reduction induced by higher intensity a-tDCS, including the reduction of facilitatory and enhancement of inhibitory intracortical mechanisms. The current findings support this assumption. ICF significantly decreased, whereas SICI and LICI were significantly enhanced at a-tDCS of ≥ 1 mA.

Although the current findings support the assumption of a-tDCS intensity and duration windows for linear effects, they also reveal that exceeding stimulation parameters beyond specific limits results in non-linear effects. Increasing stimulation intensity/duration therefore does not necessarily improve the efficacy of a-tDSC, in principle accordance with previous studies^9,14,18,20,38^.

Taken together, tDCS has been shown to induce plasticity via calcium dependent mechanisms, and at the synaptic level NMDA receptors, and GABA are involved^3,34,40,45^. In addition to reduced glutamatergic NMDA receptor activity with higher stimulation intensities, which is indicated by reduced intracortical facilitation, enhancing GABA activity^45^ might also contribute to this after-effect conversion. This is suggested by increased GABA-dependent inhibition, as shown by increased SICI, and LICI. Hereby, enhancement of inhibition regulated by both, GABA_A_-, as revealed by enhanced SICI^46^, and GABA_B_-receptor activity, as suggested by enhanced LICI^47,48^, may suggest a global enhancement of GABA activity following higher stimulation intensity. Such mechanistic concepts, however, are actually theoretical, and should be more specifically investigated in future studies.

Based on the results of the present, and other studies, with respect to mechanisms it can be assumed that within certain windows of stimulation parameters, a-tDCS induces LTP-like plasticity via calcium enhancement, supposedly driven by NMDA receptor activation, and GABA reduction. Beyond this window, enhancing stimulation intensity/duration likely results in counter-regulative mechanisms, which—as suggested by the results of the present and other studies—depend at the cellular level on calcium dynamics, and at the synaptic/neuronal network level on glutamatergic/GABAergic neurons^14,19^.

Interestingly, a secondary conversion of after-effects of tDCS with even higher stimulation intensities and duration was found in other studies (for a-tDCS:^9^, and cathodal tDCS:^22^). Respective mechanisms are not well explored, and not easily explained within the above-mentioned framework. One explanation might be that stronger, and longer stimulation will result in a calcium increase sufficient for a larger pool of neurons to develop plasticity, which would then counteract respective reversal effects.

These mechanistic explanations are however speculative at present, and should be explored more directly in future studies. In addition, it might be advantageous to add TMS-EEG in future studies, because it is a more direct measure of cortical excitability compared to TMS-induced MEPs.

## Methods

### Participants

Fifteen non-smoking healthy right-handed volunteers [8 female, mean age of 26.95 ± 6.3 (SD) years] participated in this study. The sample size was calculated (power of 0.8 and α = 0.05) based on the critical effect size generated from a pilot study on eight participants. None of the participants reported contraindications to tDCS^25^ and TMS^26^ including history of seizure, intake of CNS-acting medications, psychiatric or neurological disorders^27,28^. The study was conducted in a double-blinded crossover design with at least 7 days wash-out period between sessions^29^. Each participant was pseudo-randomly assigned to four different experimental sessions in counterbalanced order. To reduce the risk of circadian influences for each individual, all experimental sessions started at the same time of the day^30,31^. Each participant gave written informed consent before attending the study. The Human Research Ethics Committee of Monash University approved the study and we conform to the Declaration of Helsinki (1991, BMJ, 302, 1194).

### Experimental procedures

Participants were comfortably seated in a fully adjustable treatment chair (MagVenture, Denmark) with their head and arms at rest. Two pre-gelled self-adhesive Ag/AgCl electrodes (inter-electrode distance 2 cm) were placed on the right first dorsal interosseus (FDI) muscle in a belly-tendon montage to record surface electromyography (EMG). The ground electrode was placed over the styloid process of the ulna. EMG signals were filtered (bandwidth 10–500 Hz), amplified (1000 ×), and digitized at a sampling rate of 1 kHz, using a Powerlab 4/35 system (ADInstruments, Australia). MEPs were recorded using LabChart 8 software (ADInstruments, Australia) and stored on a PC for offline-analysis^19^.

### Transcranial magnetic stimulation (TMS)

TMS was applied using a MagPro R30 stimulator (MagVenture, Denmark) with a butterfly 70 mm figure-of-eight coil (max. initial dB/dt 28 KT/s near the coil surface). The coil was positioned over the left M1 with the handle pointing posterolateral. The optimal site of stimulation, which was defined as the coil position resulting in the largest MEP amplitudes elicited in the target muscle with medium TMS intensity, was marked with a soft marker as “motor hotspot”. This spot on the scalp was used for exact repositioning of the coil throughout each session. The induced current had a biphasic waveform. Resting motor threshold (RMT) was defined via the parameter estimation by sequential testing (PEST) method^32^. The current study was not conducted by aid of a neuronavigation system.

### Single-pulse TMS-induced MEPs (1 mV): CSE assessment

Twenty-five single pulse (sp)-elicited MEP amplitudes were recorded to monitor CSE before, and after intervention. The TMS intensity was adjusted as the stimulator output (%MSO) of spTMS to elicit a 1 mV peak-to-peak amplitude (SI _1 mV_) in the resting FDI on average^1,2,28^. Twenty-five single-pulse TMS (spTMS) -elicited MEPs were recorded at baseline (T_pre_), immediately (T_0_) and 30 min (T_30_) post-intervention. The mean baseline MEP was accepted if it was within the range of 1 mV ± 20%^7^. To obtain CSE changes following a-tDCS, stimulation intensity was kept constant throughout the session.

### Paired-pulse TMS-induced MEPs: intracortical excitability assessment

A paired-pulse TMS protocol (ppTMS) was used to investigate intracortical excitability, including interstimulus intervals (ISIs) of 3, 10, and 150 ms. The protocol contained 25 MEPs for short and 25 MEPs for long latency intra-cortical inhibition (SICI: 3, LICI: 150 ms), 25 MEPs for intracortical facilitation and 25 MEPs for long interval facilitation (ICF: 10 ms, LIF: 150 ms). In SICI, ICF, and LIF protocols, a subthreshold conditioning stimulus (CS: 80% of RMT) followed by a suprathreshold test stimulus (TS: SI _1 mV_)^33^ was applied. TS intensity was adjusted to achieve baseline MEPs of about 1 mV, and re-adjusted after applying a-tDCS to compensate for the effects of intervention on the MEP amplitude^34^, if required. The long interval inhibition (LICI) protocol was carried out by two consecutive identical suprathreshold CS and TS (SI _1 mV_) at an ISI of 150 ms).

### Anodal-transcranial direct current stimulation

A-tDCS was administered using a battery-driven stimulator (NeuroConn, Germany) through a pair of rubber electrodes enclosed in saline-soaked sponge pockets (5 × 7 cm; area: 35cm^2^). The selection of the stimulation parameters was based on our previous study, in which 1 mA a-tDCS reversed the excitability-enhancing effect of a-tDCS on CSE when applied for ≥ 26 min^19^. The target electrode (anode) was centered over the left M1 on the FDI hotspot, and the return electrode (cathode) over the right supraorbital area. In four pseudo-randomly ordered sessions, a-tDCS was applied with current intensities of 0.3, 0.7, 1, and 1.5 mA for 26 min. The current densities of a-tDCS under the electrodes were 0.008 (0.3 mA), 0.02 (0.7 mA), 0.029 (1 mA), and 0.04 mA/cm^2^ for 1.5 mA.

**Figure 2:**
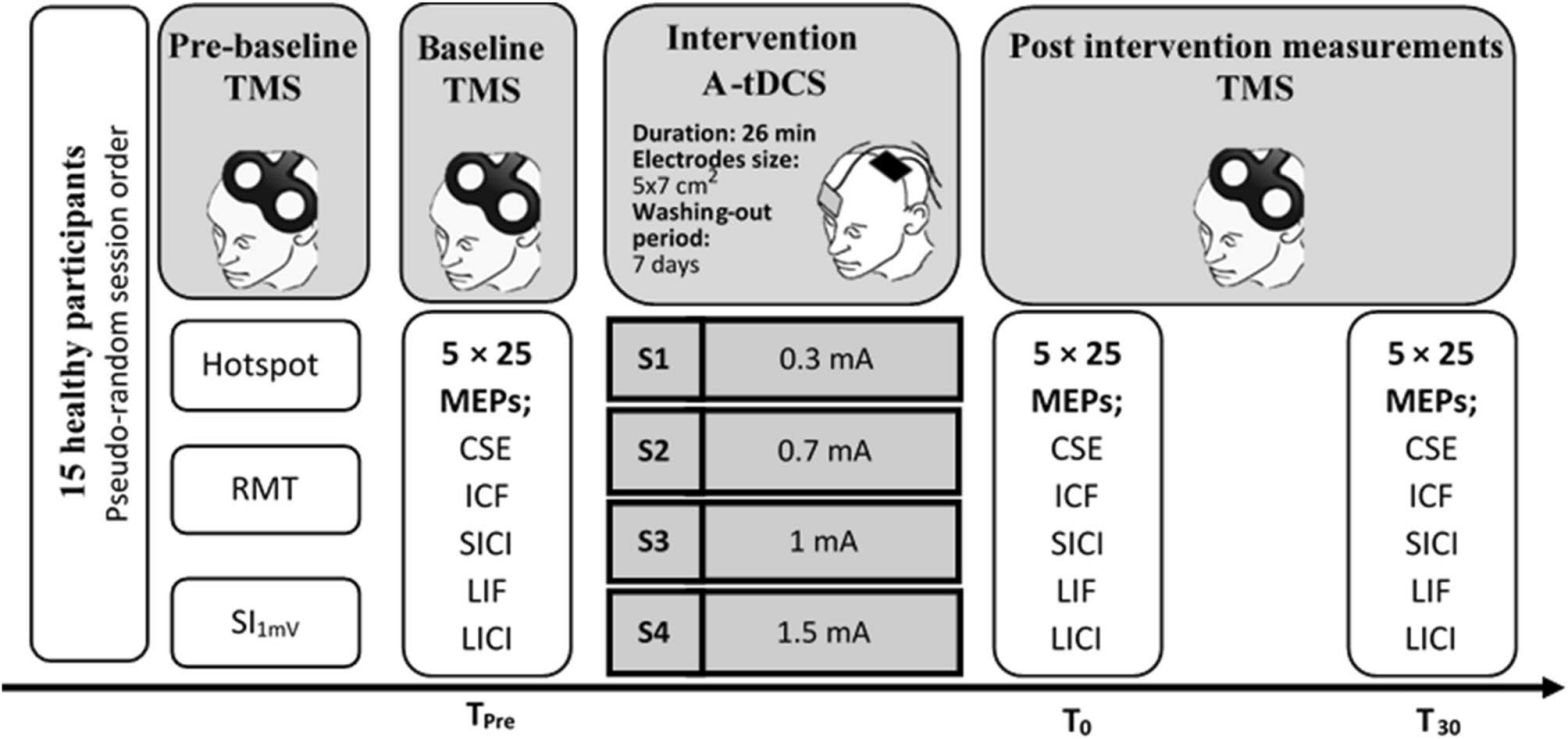
Schematic representation of the experimental procedure for each session. The time course is from left to right. *TMS* transcranial magnetic stimulation, *S* session, *MEPs* motor evoked potentials, *CSE* corticospinal excitability, *ICF* intra-cortical facilitation, *LICI* long interval intra-cortical inhibition, *SICI* short latency intra-cortical inhibition, *LIF* long interval facilitation, *A-tDCS* anodal-transcranial direct current stimulation, *RMT* resting motor threshold, *SI*_*1mV*_ stimulator intensity required for a peak-to-peak MEP amplitude of approximately 1 mV.

To minimize any potential discomfort, a 15 s ramp-up/down was applied at the beginning and end of the stimulation. During stimulation, participants were instructed to remain relaxed and keep their hands in a relaxed position. The experimental design of the current study is summarised in Fig. 2.

### Measurement of the side and adverse effects

Side effects were recorded at the beginning, middle and end of the stimulation in all experimental sessions. All participants were asked to complete a form to record the side and adverse effects of a-tDCS. The form contained rating scales for the presence and severity of common side effects such as itching, tingling, and burning sensation under the electrodes^35,36^and other adverse effects including headache and pain during and after stimulation (Brunoni et al.^25^). The unpleasantness of any scalp sensation was rated via numeric analogue scales (NAS) (i.e. 0 = no feeling to 10 = worst imaginable sensation). To test the efficacy of blinding, participants were asked at the end of each session to tell if they did perceive any difference between the present stimulation compared to previous session(s). They answered choosing 'Yes', 'No'.

### Statistical analyses

The peak-to-peak amplitudes of 25 single-pulse MEPs (sp-MEPs) were calculated and averaged for the three time points (T_pre_, T_0_, and T_30_). The size of each conditioned MEP was expressed as percentage of the unconditioned test MEPs for paired-pulse MEPs (pp-MEPs). The means of SICI, LICI, ICF, and LIF (each 25 MEPs) were calculated for each time point separately. A one-way repeated measures ANOVA (rmANOVA) was used on baseline values to rule out carry over effects between the experimental conditions. The Shapiro–Wilk test was applied to explore the normality of each outcome. Separate repeated measure (rm) ANOVAs were calculated to assess how main effects of ‘intensity’ with 4 levels (0.3, 0.7, 1, and 1.5), and ‘time’ with 3 levels (T_pre_, T_0_, and T_30_) and the interaction between the effects may have affected CSE, SICI, LICI, ICF, and LIF. Mauchly’s test was used to assess the validity of the sphericity assumption for the rmANOVAs. Greenhouse–Geisser-corrections were applied for non-spherical data^37^. If a significant effect was found, Bonferroni-corrected post hoc paired-sample t tests were conducted to explore whether the baseline value of each experimental condition differed significantly from post-intervention time points (T_0_ and T_30_).

To assess whether participants were successfully blinded to the experimental conditions, Pearson’s chi-square test was used. In addition, a one-way ANOVA was carried out on the mean scores recorded in the numerical analogue scale ratings to assess differences of side effects between sessions. Statistical analyses were performed using SPSS 25 (IBM, NY, USA) with a level of significance of P = 0.05 for all statistical tests. All results are presented as mean ± standard error of means (SEM).

### Suggestions for future studies

It would be interesting to monitor excitability measures also during stimulation, to receive more profound knowledge about the temporal dynamics of the development of plasticity by tDCS. Furthermore, additional experiments should be conducted to add mechanistic information via more direct exploration of the contribution of ion channels and neurotransmitters to the effects of stimulation, which might also require animal experiments. Coil positioning was performed manually based on physiological data without the aid of a neuronavigation system. This procedure might result in larger MEP variability, as compared to a navigation-supported procedure, which should be taken into consideration for future studies. Moreover, the intensity threshold of M1 a-tDCS should be studied in older adults and patients with neurological disorders to enhance the transferability of the findings, which might be relevant for clinical application of this intervention. Studies using behavioural outcome measures would provide valuable information to investigate if the reversal of CSE effects revealed in the present study also affects motor or cognitive behaviour accordingly.

## Conclusions

The results of this study show that increasing the intensity of a-tDCS does not necessarily enhance its efficacy to induce LTP-like plasticity, but might even reverse the direction of the effects, especially for specific prolonged stimulation durations, and that this conversion critically depends on the intensity of stimulation. Moreover, the results show that respective corticospinal effects are mirrored at the level of intracortical circuits.

## References

[1] Nitsche MA, Paulus W (2001). Sustained excitability elevations induced by transcranial DC motor cortex stimulation in humans. Neurology.

[2] Nitsche MA, Fricke K, Henschke U, Schlitterlau A, Liebetanz D, Lang N, Henning S, Tergau F, Paulus W (2003). Pharmacological modulation of cortical excitability shifts induced by transcranial direct current stimulation in humans. J. Physiol..

[3] Wiethoff S, Hamada M, Rothwell JC (2014). Variability in response to transcranial direct current stimulation of the motor cortex. Brain Stimul..

[4] Nitsche MA, Paulus W (2000). Excitability changes induced in the human motor cortex by weak transcranial direct current stimulation. J. Physiol..

[5] Tremblay S, Beaule V, Lepage J-F, Theoret H (2013). Anodal transcranial direct current stimulation modulates GABAB-related intracortical inhibition in the M1 of healthy individuals. NeuroReport.

[6] Strube W, Bunse T, Malchow B, Hasan A (2015). Efficacy and interindividual variability in motor-cortex plasticity following anodal tDCS and paired-associative stimulation. Neural Plast..

[7] Labruna L (2016). Efficacy of anodal transcranial direct current stimulation is related to sensitivity to transcranial magnetic stimulation. Brain Stimul..

[8] Jamil A (2016). Systematic evaluation of the impact of stimulation intensity on neuroplastic after-effects induced by transcranial direct current stimulation. J. Physiol..

[9] Agboada D, Mosayebi Samani M, Jamil A, Kuo MF, Nitsche MA (2019). Expanding the parameter space of anodal transcranial direct current stimulation of the primary motor cortex. Sci. Rep..

[10] Pellicciari MC, Brignani D, Miniussi C (2013). Excitability modulation of the motor system induced by transcranial direct current stimulation: A multimodal approach. NeuroImage..

[11] Romero Lauro LJ (2014). TDCS increases cortical excitability: Direct evidence from TMS-EEG. Cortex..

[12] Romero Lauro LJ, Pisoni A, Rosanova M, Casarotto S, Mattavelli G, Bolognini N, Vallar G (2016). Localizing the effects of anodal tDCS at the level of cortical sources: a reply to Bailey et al. Cortex.

[13] Pisoni A, Mattavelli G, Papagno C, Rosanova M, Casali A, Lauro LJ (2018). Cognitive enhancement induced by anodal tDCS drives circuit-specific cortical plasticity. Cereb. Cortex.

[14] Monte-Silva K (2013). Induction of late LTP-like plasticity in the human motor cortex by repeated noninvasive brain stimulation. Brain Stimul..

[15] Batsikadze G, Moliadze V, Paulus W, Kuo MF, Nitsche MA (2013). Partially non-linear stimulation intensity-dependent effects of direct current stimulation on motor cortex excitability in humans. J. Physiol..

[16] Lopez-Alonso V, Cheeran B, Rio-Rodriguez D, Fernandez-Del-Olmo M (2014). Inter-individual variability in response to non-invasive brain stimulation paradigms. Brain Stimul..

[17] Tremblay S (2016). Systematic assessment of duration and intensity of anodal transcranial direct current stimulation on primary motor cortex excitability. Eur. J. Neurosci..

[18] Vignaud P, Mondino M, Poulet E, Palm U, Brunelin J (2018). Duration but not intensity influences transcranial direct current stimulation (tDCS) after-effects on cortical excitability. Neurophysiol. Clin..

[19] Hassanzahraee M, Nitsche AM, Zoghi M, Jaberzadeh S (2020). Determination of anodal tDCS duration threshold for reversal of corticospinal excitability: An investigation for induction of counter-regulatory mechanisms. Brain Stimul..

[20] Kidgell DJ, Daly RM, Young K, Lum J, Tooley G, Jaberzadeh S, Zoghi M, Pearce AJ (2013). Different current intensities of anodal transcranial direct current stimulation do not differentially modulate motor cortex plasticity. Neural Plast..

[21] Chew T, Ho K-A, Loo CK (2015). Inter- and intra-individual variability in response to transcranial direct current stimulation (tDCS) at varying current intensities. Brain Stimul..

[22] Mosayebi Samani M, Agboada D, Jamil A, Kuo MF, Nitsche MA (2019). Titrating the neuroplastic effects of cathodal transcranial direct current stimulation (tDCS) over the primary motor cortex. Cortex.

[23] Lisman JE, Zhabotinsky AM (2001). A model of synaptic memory: A CaMKII/PP1 switch that potentiates transmission by organizing an AMPA receptor anchoring assembly. Neuron.

[24] Misonou H (2004). Regulation of ion channel localization and phosphorylation by neuronal activity. Nat. Neurosci..

[25] Brunoni AR, Amadera J, Berbel B, Volz MS, Rizzerio BG, Fregni F (2011). A systematic review on reporting and assessment of adverse effects associated with transcranial direct current stimulation. Int. J. Neuropsychopharmacol..

[26] Wassermann EM (1998). Risk and safety of repetitive transcranial magnetic stimulation: Report and suggested guidelines from the International Workshop on the Safety of Repetitive Transcranial Magnetic Stimulation, June 5–7, 1996. Electroencephalogr. Clin. Neurophysiol..

[27] Nitsche MA (2008). Transcranial direct current stimulation: State of the art 2008. Brain Stimul..

[28] Rossini PM (2015). Non-invasive electrical and magnetic stimulation of the brain, spinal cord, roots and peripheral nerves: Basic principles and procedures for routine clinical and research application. Clin. Neurophysiol..

[29] Woods AJ (2016). A technical guide to tDCS, and related non-invasive brain stimulation tools. Clin. Neurophysiol..

[30] Krause B, Cohen Kadosh R (2014). Not all brains are created equal: The relevance of individual differences in responsiveness to transcranial electrical stimulation. Front. Syst. Neurosci..

[31] Li LM, Uehara K, Hanakawa T (2015). The contribution of interindividual factors to variability of response in transcranial direct current stimulation studies. Front. Cell Neurosci..

[32] Mishory A (2004). The maximum-likelihood strategy for determining transcranial magnetic stimulation motor threshold using parameter estimation by sequential testing is faster than conventional methods with similar precision. J ECT..

[33] Kujirai T (1993). Corticocortical inhibition in human motor cortex. J. Physiol..

[34] Nitsche MA (2005). Modulating parameters of excitability during and after transcranial direct current stimulation of the human motor cortex. J. Physiol..

[35] Poreisz C, Boros K, Antal A, Paulus W (2007). Safety aspects of transcranial direct current stimulation concerning healthy subjects and patients. Brain Res. Bull..

[36] George MS, Aston-Jones G (2010). Noninvasive techniques for probing neurocircuitry and treating illness: Vagus nerve stimulation (VNS), transcranial magnetic stimulation (TMS) and transcranial direct current stimulation (tDCS). Neuropsychopharmacology.

[37] Meyers LS, Gamst G, Guarino AJ (2006). Applied Multivariate Research: Design and Interpretation.

[38] Bastani A, Jaberzadeh S (2013). Differential modulation of corticospinal excitability by different current densities of anodal transcranial direct current stimulation. PLoS One.

[39] Hummel F (2005). Effects of non-invasive cortical stimulation on skilled motor function in chronic stroke. Brain.

[40] Stagg CJ, Nitsche MA (2011). Physiological basis of transcranial direct current stimulation. Neuroscientist.

[41] Nitsche MA (2004). GABAergic modulation of DC stimulation-induced motor cortex excitability shifts in humans. Eur. J. Neurosci..

[42] Ziemann U, Rothwell JC, Ridding MC (1996). Interaction between intracortical inhibition and facilitation in human motor cortex. J. Physiol. (Lond)..

[43] Ziemann U, Chen R, Cohen LG, Hallett M (1998). Dextromethorphan decreases the excitability of the human motor cortex. Neurology.

[44] Chen R, Tam A, Bütefisch C, Corwell B, Ziemann U, Rothwell JC, Cohen LG (1998). Intracortical inhibition and facilitation in different representations of the human motor cortex. J. Neurophysiol..

[45] Stagg CJ (2009). Polarity-sensitive modulation of cortical neurotransmitters by transcranial stimulation. J. Neurosci..

[46] Stagg CJ (2011). Relationship between physiological measures of excitability and levels of glutamate and GABA in the human motor cortex. J. Physiol..

[47] Sanger TD, Garg RR, Chen R (2001). Interactions between two different inhibitory systems in the human motor cortex. J. Physiol..

[48] McDonnell MN, Orekhov Y, Ziemann U (2006). The role of GABA(B) receptors in intracortical inhibition in the human motor cortex. Exp. Brain Res..

